# Abnormal Behavior in Cascading Dynamics with Node Weight

**DOI:** 10.1371/journal.pone.0139941

**Published:** 2015-10-09

**Authors:** Jianwei Wang, Lin Cai, Bo Xu, Yuedan Wu

**Affiliations:** School of Business Administration, Northeastern University, Shenyang 110819, P. R. China; Beihang University, CHINA

## Abstract

Considering a preferential selection mechanism of load destination, we introduce a new method to quantify initial load distribution and subsequently construct a simple cascading model. By attacking the node with the highest load, we investigate the cascading dynamics in some synthetic networks. Surprisingly, we observe that for several networks of different structural patterns, a counterintuitive phenomenon emerges if the highest load attack is applied to the system, i.e., investing more resources to protect every node in a network inversely makes the whole network more vulnerable. We explain this ability paradox by analyzing the micro-structural components of the underlying network and therefore reveals how specific structural patterns may influence the cascading dynamics. We discover that the robustness of the network oscillates as the capacity of each node increases. The conclusion of the paper may shed lights on future investigations to avoid the demonstrated ability paradox and subsequent cascading failures in real-world networks.

## Introduction

The investigation of the network robustness is a vital part of many fields in the academia, and especially more and more researchers are concerned about the the vulnerability of natural and man-made complex systems under cascading failures caused by attacking some key nodes or edges. Cascading failures occur in many infrastructure networks, for example, large-scale blackouts of power grids [1–5], severe traffic jams in traffic networks [6], and the Internet collapse caused by congestion [7]. In these networks, the loads exist in forms of the electric charge, traffic flows, or data flows. In general, the network system is in the maintenance of normal and efficient operations. However, once some key nodes or edges fail, it triggers the redistribution of the load of the failed nodes or edges, which may further lead to the successive failures of many other parts of the entire network. This step-by-step process is what we call the cascading failure.

To minimize the impact of cascading failures at the global system level and better protect many infrastructure networks, by analyzing the dynamic mechanism of cascading failures, a number of important aspects of cascading failures have been discussed and many valuable results have been obtained, including the models for describing the cascade phenomena [8–14], the cascade on the epidemic spreading [15–18], the cascading mitigation strategies [19–26], the percolation in multiplex networks [27, 28], the cascading phenomenon in game dynamics [29], the efficiency of random or intentional attacks [30–34], the percolation in interdependent networks [35–43], and so on. In all cited studies above, the initial distribution of load on nodes or edges and the definition of redistribution rules of the load from invalid nodes or edges are of vital importance. In previous works, the initial load on a node or an edge is generally estimated by its global betweenness, and the load will be recalculated according to betweenness centrality if some nodes or edges are removed. Although the betweenness method can be widely applied to the cascading model, it is still a debatable issue whether this method can better quantify the flow of physical quantities in networks, since it fails to consider the heterogeneous degree distributions of most real-world networks.

To this end, by analyzing the dynamic characteristics of cascading failure, we summarize two deficiencies of the traditional betweenness method in modeling cascading failures: **1**. It ignores the information of the node weight; **2**. It fails to consider the fact that the load generated from a node or an edge may preferentially select destinations in realistic networks. In this paper, We propose a new method to quantify initial load distributions by integrating the information of node degree and the mechanism of preferential destination selection. To the best of our knowledge, this is the first study to consider the revised betweenness method from the perspectives of the load characteristics. Moreover, different from previous studies, we pose a key question: is there an inevitable positive correlation between the total capacity of all nodes and the network robustness? To address this problem, by removing the single node with the highest load, we focus on the correlation between the capacity parameter and the network robustness in some synthetic networks. We surprisingly observe a counterintuitive phenomenon which is termed as “ability paradox” in this research, i.e., in some networks with specific micro-structural patterns, stronger capacity inhibits the emergence of the largest connected component and thus decreases the network robustness against cascading failures. By carefully analyzing redistribution of the load during the cascading process, as the total capacity of a network increases, the abnormal oscillation of several quantitative indexes reflecting the network robustness can be explained by the ring structure in the remaining network after removing a node. When the capacity parameter increases, paradoxically, the revivals of some nodes may trigger the failures of more nodes. This explains why investing more police forces in traffic networks inversely makes more jams in traffic flows. Our findings have profound implications for preventing and mitigating various cascading-failure-induced disasters in the real world.

## The model

**Fig 1:**
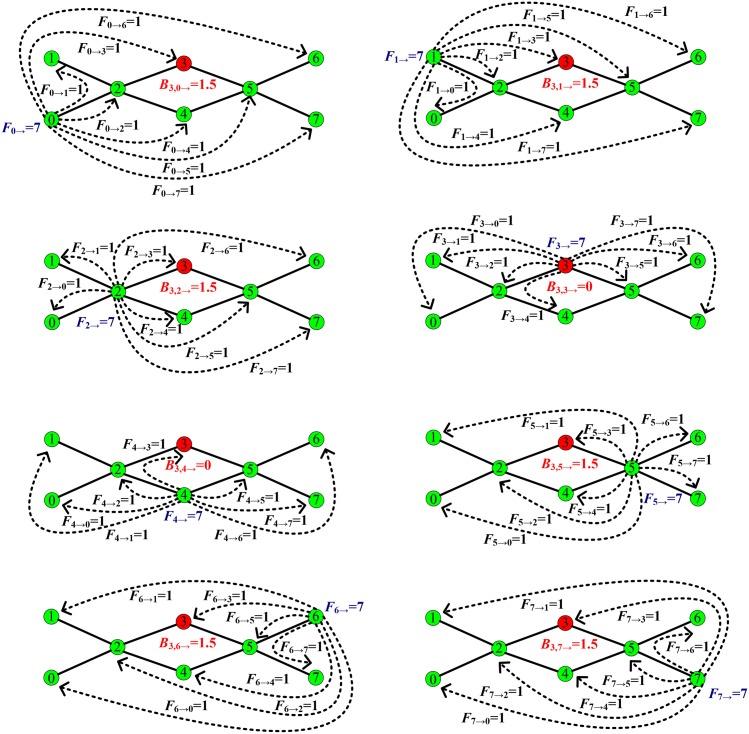
The calculation of the load transported by a node in previous work. In the betweenness method, assume that the load was transmitted along the shortest path between every pair of nodes. If calculating the initial load on a node, we need to count the effect of the load transmitted between all pairs of nodes on this node. For example, we calculate the load on node 3. We use *F*_*i* → *j*_ and *B*_*m*, *i* →_ to denote the load transported from node *i* to *j* and the load passing through node *m* in all load generated by node *i*, respectively. By calculating the load passing through node 3 and generated by every node, we get *B*_3,0 →_ = 1.5, *B*_3,1 →_ = 1.5, *B*_3,2 →_ = 1.5, *B*_3,3 →_ = 0, *B*_3,4 →_ = 1.5, *B*_3,5 →_ = 1.5, *B*_3,6 →_ = 1.5, and *B*_3,7 →_ = 1.5. Therefore, we can obtain *B*_3_ = 9, which represents the total load transported by node 3.

**Fig 2:**
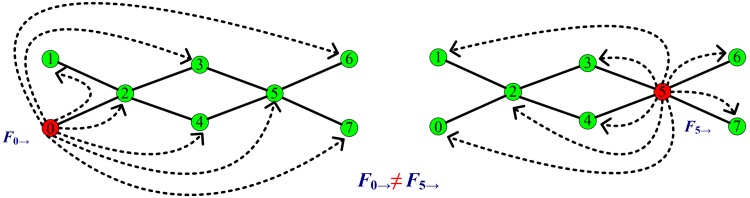
Node degree influences the number of the load generated by this node. In the Internet, traffic networks, and the power grid, in general, the bigger the degree of a node, the higher the load generated by it. We define *F*_*i* →_ to represent the total load generated by node *i*, which is transported to other nodes in a network. Owing to the effect of the node degree, *F*_0 →_ should be not equal to *F*_5 →_.

In previous works on cascading failures, for a given network, many researchers suppose that at each time step one unit of the relevant quantity, which can be information, energy, etc., is exchanged between every pair of nodes and transmitted along the shortest path connecting them. If there is more than one shortest path connecting two given nodes, the packet is divided evenly at each branching point. Therefore, in many previous cascading models based on the betweenness, the initial load is naturally assigned to the total number of shortest paths passing through the node (see Fig 1). Although the classic betweenness method is widely applied to the cascading modeling, it ignores the heterogeneous impact of the degree-based node weight on the load generated from the given node. (see Fig 2). In Fig 2, owing to the effect of the node degree, at each time step, the load *F*_0 →_ generated by node 0 should be not equal to the load *F*_5 →_ generated by node 0.

In real networks, in general, the bigger the degree of a node, the higher the load generated from it. To better quantify the effect of the degree on the initial load, we revise the classic betweenness measurement. Firstly, we assume the weight of node *i* in a network to be $\varpi_{i}=k_{i}^{\alpha }$, where *α* is a tunable weight parameter, governing the strength of the node weight. We use *F*_*i* →_ to denote the load generated by node *i* at each time step. In our model, we simply assume *F*_*i* →_ = *ϖ*_*i*_. Next a natural question arises: how much load in *F*_*i* →_ is transported to node *j*? In real networks, nodes with higher weight usually receives more load from others. Therefore, we propose a preferential destination selection principle by assuming that the loads transmitted from node *i* to node *j* is proportional to the weight of node *j*, i.e.,

$$\begin{array}{c} F_{i\to j}=F_{i\to }\frac{\varpi_{j}}{\sum_{m\in V}\varpi_{m}-\varpi_{i}}, \end{array}$$ (1)

where *V* is the set of nodes. Similarly, the loads transmitted from node *j* to node *i* is

$$\begin{array}{c} F_{j\to i}=F_{j\to }\frac{\varpi_{i}}{\sum_{m\in V}\varpi_{m}-\varpi_{j}}. \end{array}$$ (2)

**Fig 3:**
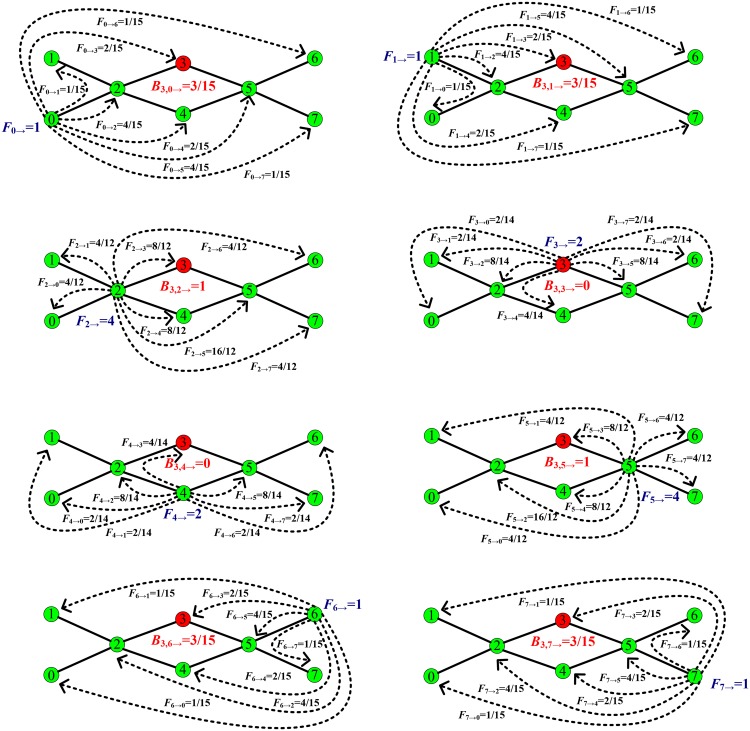
The scheme illustrates the calculating process of the initial load on a node in our new method. Compared with Fig 1, we here calculate the initial load on node 3. When *α* = 1, We can get that the loads generated by nodes 0, 1, 2, 3, 4, 5, 6, and 7 are *F*_0_ = 1, *F*_1_ = 1, *F*_2_ = 4, *F*_3_ = 2, *F*_4_ = 2, *F*_5_ = 4, *F*_6_ = 1, and *F*_7_ = 1, respectively. According to the preferential principle of the destination selection of the load transported, we can calculate the load exchanged between any two nodes. We can further get the loads passing through node 3 in the loads generated by every node are *B*_3,0 →_ = 3/15, *B*_3,1 →_ = 3/15, *B*_3,2 →_ = 1, *B*_3,3 →_ = 0, *B*_3,4 →_ = 0, *B*_3,5 →_ = 1, *B*_3,6 →_ = 3/15, and *B*_3,7 →_ = 3/15, respectively. Therefore, we can obtain that the total load *B*_3_ transported by node 3 is 2.8, i.e., the initial load *L*_3_ on node 3 is 2.8.

Here, in general, *F*_*i* → *j*_ ≠ *F*_*j* → *i*_. Loads *F*_*i* → *j*_ are transmitted along the shortest paths connecting *i* and *j*. If there are more than one shortest path connecting the two given nodes, the loads transported are divided evenly at each branching point. Let $\rho_{k}^{\left(i,j\right)}$ denote the contribution of one unit of a physical quantity transmitted between the ordered pair (*i*, *j*) to the load on *k*. Then, the overall contribution of the load transmitted between the ordered pair (*i*, *j*) to the load on *k* is $L_{k}^{\left(i,j\right)}=(F_{i\to j}+F_{j\to i})\rho_{k}^{\left(i,j\right)}$. The initial load *L*_*k*_ on node *k* is then

$$\begin{array}{c} L_{k}=\sum_{i,j}L_{k}^{\left(i,j\right)}, \end{array}$$ (3)

where the sum is over all pairs (*i*, *j*) of nodes in a network (see Fig 3). When *α* = 0, the initial load on a node is equal to the total number of shortest paths passing through the node, in agreement with previous classic betweenness method [21, 34].

Each node *i* is assigned to have a finite capacity *C*_*i*_, i.e., the maximum load that node can handle. In man-made networks, the capacity is severely limited by cost. Thus, it is natural to assume that the capacity *C*_*i*_ of node *i* is proportional to its initial load *L*_*i*_,

$$\begin{array}{c} C_{i}=\left(1+\beta \right)L_{i},\;\;\;\;\;\;\;\;\;i=1,2,...,N, \end{array}$$ (4)

where the constant *β* ≥ 0 is the tolerance parameter. Node *i* maintains its normal and efficient function if its load is less than or equal to its capacity; otherwise it fails and is removed from the network. The removal of nodes, in general, leads to a global redistribution of shortest paths. The loads on some particular nodes can then change and may become larger than their capacities. All the overloaded nodes are removed simultaneously from the network, which leads to a new redistribution of loads and subsequent overloads may occur. The cascading propagation will stop when the loads on all remaining nodes do not exceed their capacities.

## The analysis of the cascading model

Here we focus on global cascades triggered by attacking one node with the highest initial load. The damage is quantified by two measures, i.e., the number *G*_*N*_ of nodes in the largest connected component and the avalanche size *S*. Two fundamental questions are, how the parameter *α* affect the network robustness against cascading failure and whether there exists a positive relationship between the parameter beta and the robustness of the network. To answer these questions, we investigate the cascading dynamics in five synthetic networks.

**Fig 4:**
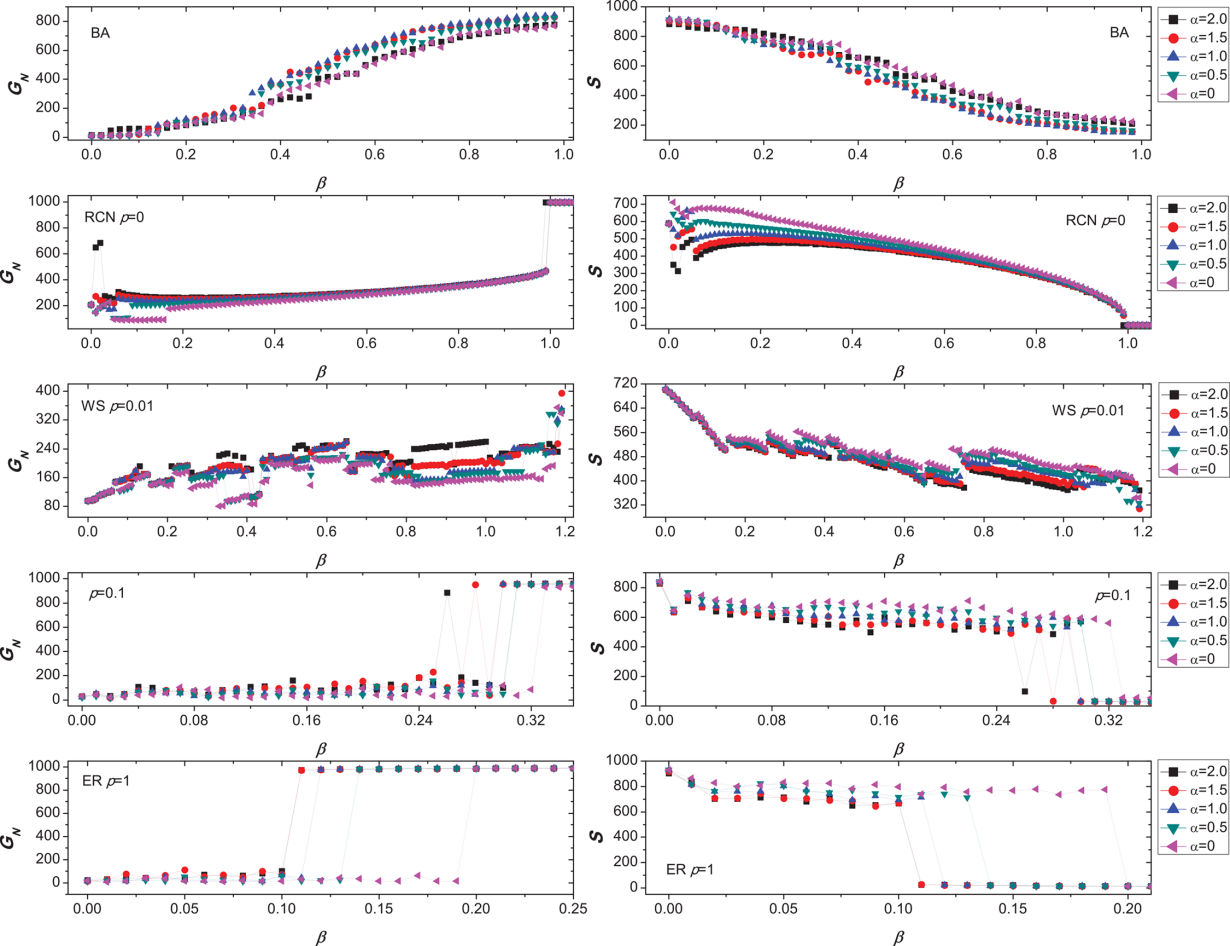
Dynamics of cascading failures in five synthetic networks, *N* = 1000, ⟨*k*⟩ = 4. (a) The scale-free networks were constructed by BA model [44] and data is averaged over 20 independent runs of node removal. (b) Ring-coupled network. (c) The WS small world network [45] created by a ring-coupled network with the rewiring *p* = 0.01. (d) Synthetic network constructed by a ring-coupled network with the rewiring *p* = 0.1. (e) Random network created by a ring-coupled network with the rewiring *p* = 1. (b-e) Data is from a single run. (a-e) We plot *G* and *S* as functions of the parameter *β* for five cases of *α* = 0, *α* = 0.5, *α* = 1, *α* = 1.5, and *α* = 2.

In Fig 4, We plot *G*_*N*_ and *S* as functions of the parameter *β*. (a) It is natural that, the bigger the value *β*, the more the number *G*_*N*_ of nodes in the largest connected component, and the smaller the number *S* of the failed nodes induced by removing the node with the highest load, in scale-free networks constructed by BA model [44]. (b) In a ring network, surprisingly, we see an unexpected behavior: as *β* increases, all curves show an obvious and wide fluctuation, which means that sometimes the network robustness decreases against cascading failures. For example, *G*_*N*_ is 685 when *β* = 0.02, however, *G*_*N*_ becomes 454 when *β* = 0.98, in the case of *α* = 2. (c) We difficultly obtain the correlation between the WS network robustness and the capacity parameter *β*. Similar to (b), the capacity enhancement of every node may not be able to improve the robustness of the whole network. And the range of the wave curves is larger than that of (b). When investing more resources to protecting the network, the uncertainty of the network robustness causes a lot of trouble to managers. (d) When the rewiring probability *p* = 0.1, we also observe that, as *β* increases, both *G*_*N*_ and *S* bounces up and down in the smaller range of *β* (0.25 < *β* < 0.3). (e) When *p* = 1, the generated networks are same as the ER networks. As *β* increases, the oscillation is almost smaller. In addition, we also find that the parameter *α* has almost no effect on the network robustness against cascading failure.

**Fig 5:**
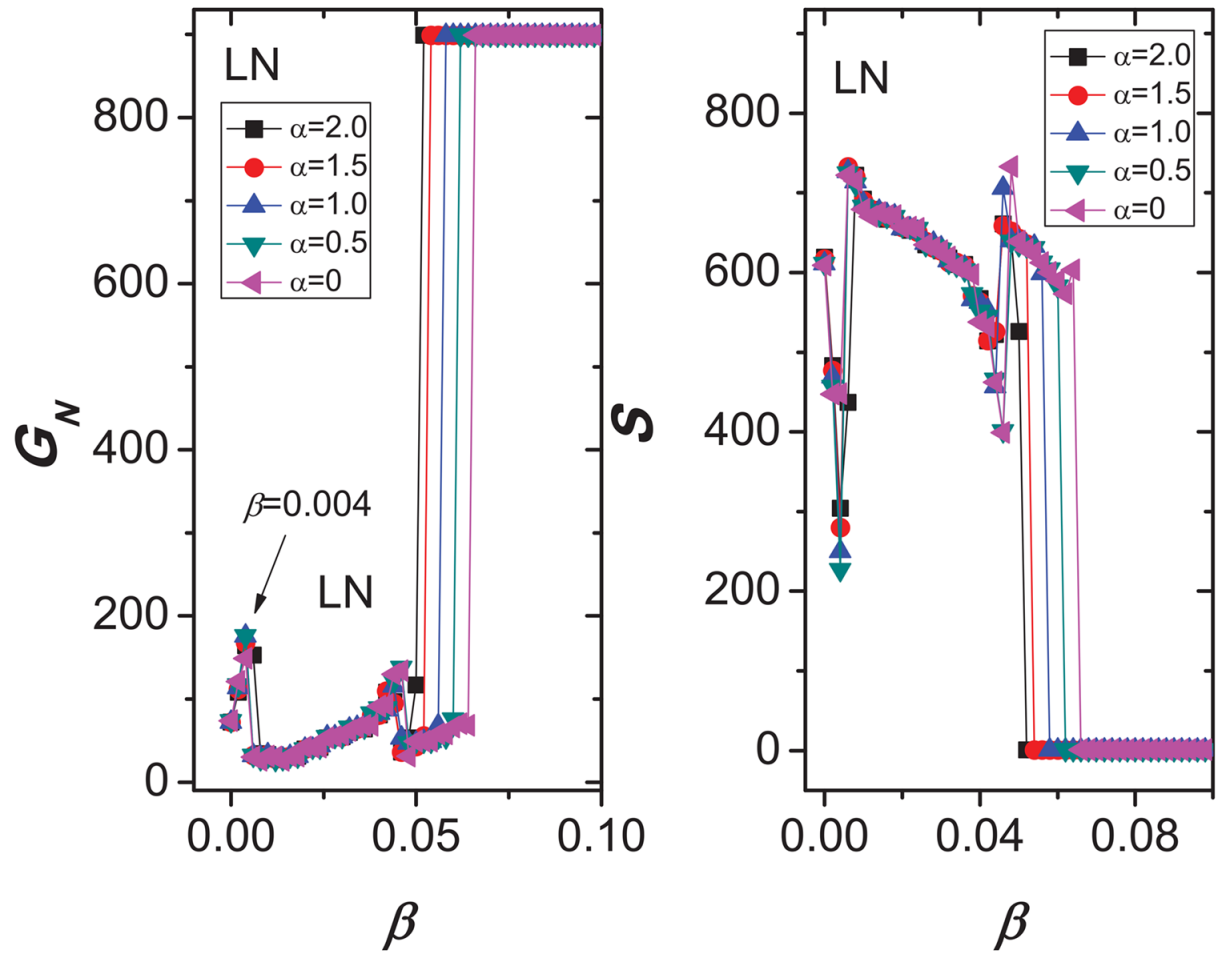
Demonstration of cascading failures in a lattice network with 900 nodes and 1740 edges.

The ability paradox observed in the variety of structures is sufficient to determine the universal oscillation characteristics in cascading dynamics. For better explaining the ability paradox in cascading model, in Fig 5 we also explore the cascading dynamics in a lattice network with the simple topological structure, which have 900 nodes (30 × 30) and 1740 edges. In Fig 5, as value *β* increases from 0.004 to 0.05, we clearly observe that the number *G*_*N*_ of nodes in the largest connected component decreases sharply, then slowly increases. For example, when *β* = 0.004, the values of *G*_*N*_ in five cases of *α* = 0, *α* = 0.5, *α* = 1.0, *α* = 1.5, and *α* = 2.0, are 149, 176, 176, 168, and 164, respectively. However, when *β* = 0.006, the values of *G*_*N*_ in five cases of *α*, are 30, 32, 32, 32, and 153, respectively. As value *β* increases from 0.004 to 0.05, the avalanche size *S* increases sharply, then slowly decreases. Although this ability paradox can be observed in some networks, it is very difficult to understand this counterintuitive phenomenon, i.e., increasing protection investment leads to reduced network robustness.

**Fig 6:**
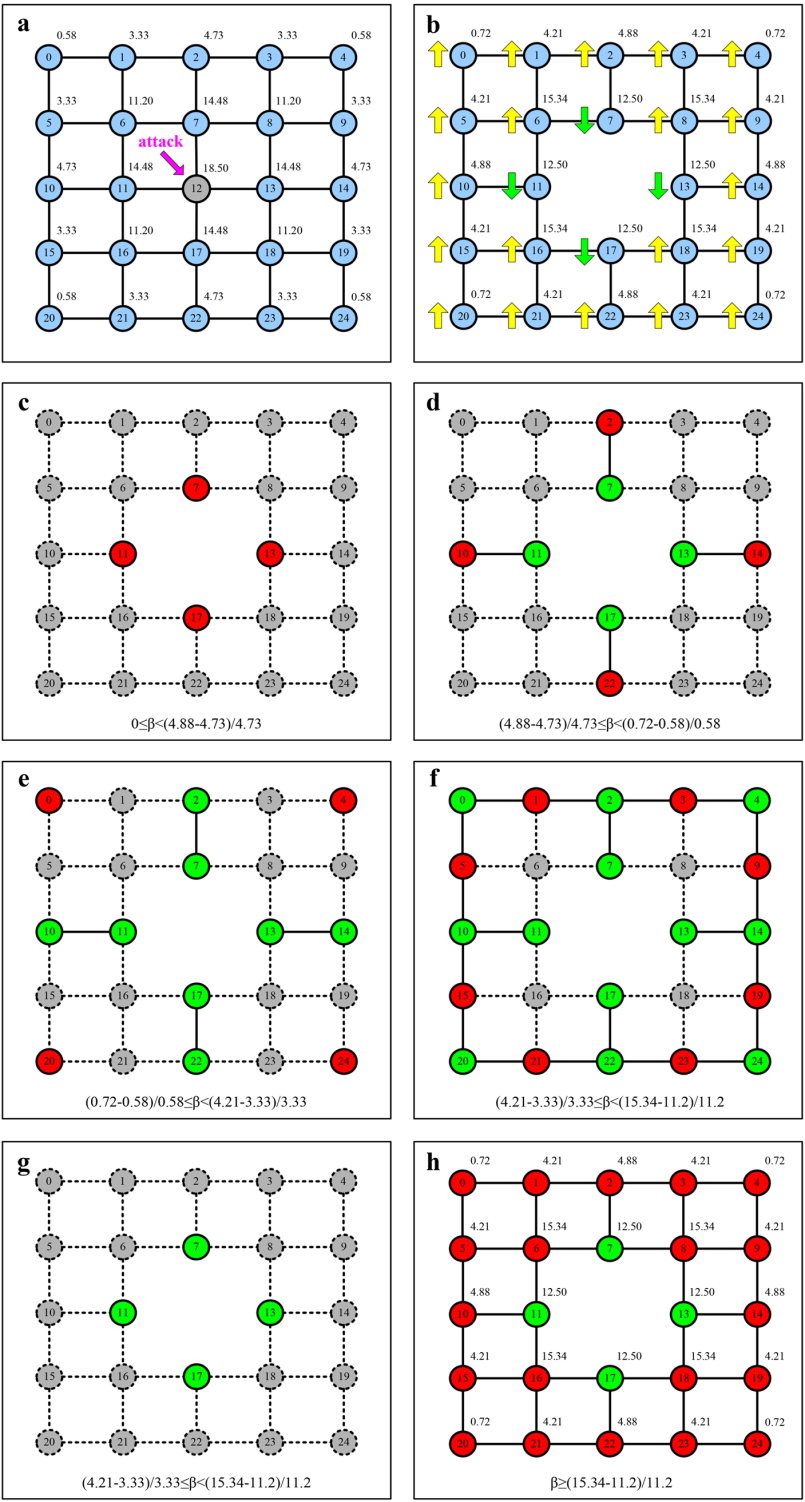
A series of steps in cascading propagation on a simple lattice network by setting *α* = 1.

Why does the enhancement of the capacities inversely reduce the network robustness against cascading failures? To help answer this question we apply a lattice network with the smaller size (5 × 5) to step by step simulate the cascading process and examine a series of steps in it. In Fig 6, (a) At the initial stage, by setting *α* = 1, we calculate the initial load on each node and remove the node 12 with the highest load, where the numbers inside and outside the blue solid line circle denote the labels of nodes and the initial load on each node. (b) After node 12 is removed (four edges connected to node 12 also are removed), we recalculate the load (the numbers outside the blue solid line circle) on each node in the remaining network and label the fluctuation of the load on each remaining node by arrows, of which the yellow arrow up denotes that the load on a node increases than its initial load, and the green arrow down denotes that the load on a node decreases than its initial load. We observe that four nodes linked to node 12 (i.e.,nodes 7, 11, 13, and 17) had the lower load than its initial load, while the load on the other nodes inversely increase. Next, according to the different ranges of the values of *β*, we examine step by step the cascading propagation. (c) We use the red solid line circle and the grey dotted line circle to represent the surviving and failed nodes, respectively. And the dotted straight line between two nodes denotes the failed edges. When 0 ≤ *β* < (4.88 − 4.73)/4.73, consequently, only four nodes (7, 11, 13, and 17) survive because the loads on them are smaller than their capacities after node 12 fails. But the number *G*_*N*_ in the largest connected component is 0 because all four nodes are isolated. (d) By increasing the value of *β* gradually, we observe the change of the number *G*_*N*_. Since the fluctuation of the load on nodes 2, 10, 14 and 22 is smallest, when the value of *β* is in the range of (4.88 − 4.73)/4.73 ≤ *β* < (0.72 − 0.58)/0.58, these four nodes revive and the value of *G*_*N*_ is 2. (e) When (0.72 − 0.58)/0.58 ≤ *β* < (4.21 − 3.33)/3.33, although nodes 0, 4, 20, and 24 revive, the value of *G*_*N*_ is still 2 for the reason that these nodes are isolated as well. (f) When (4.21 − 3.33)/3.33 ≤ *β* < (15.34 − 11.22)/11.22, nodes 1, 3, 5, 9, 15, 19, 21, and 23 revive and form quickly a ring structure all together the nodes represented by the red and the green solid line in (e). (g) We recalculate the load on the remaining nodes again. The results indicate that the load on these nodes exceeds their endurance capacities except 7,11,13 and 17. Thus, these nodes will fail and are removed from the network. But, the value of *G*_*N*_ is 0, owing to nodes 7, 11, 13, and 17 are isolated. (h) Eventually, when the value of *β* increases to (15.34 − 11.2)/11.2, all nodes revive and the number *G*_*N*_ of the largest connect component is 24.

**Fig 7:**
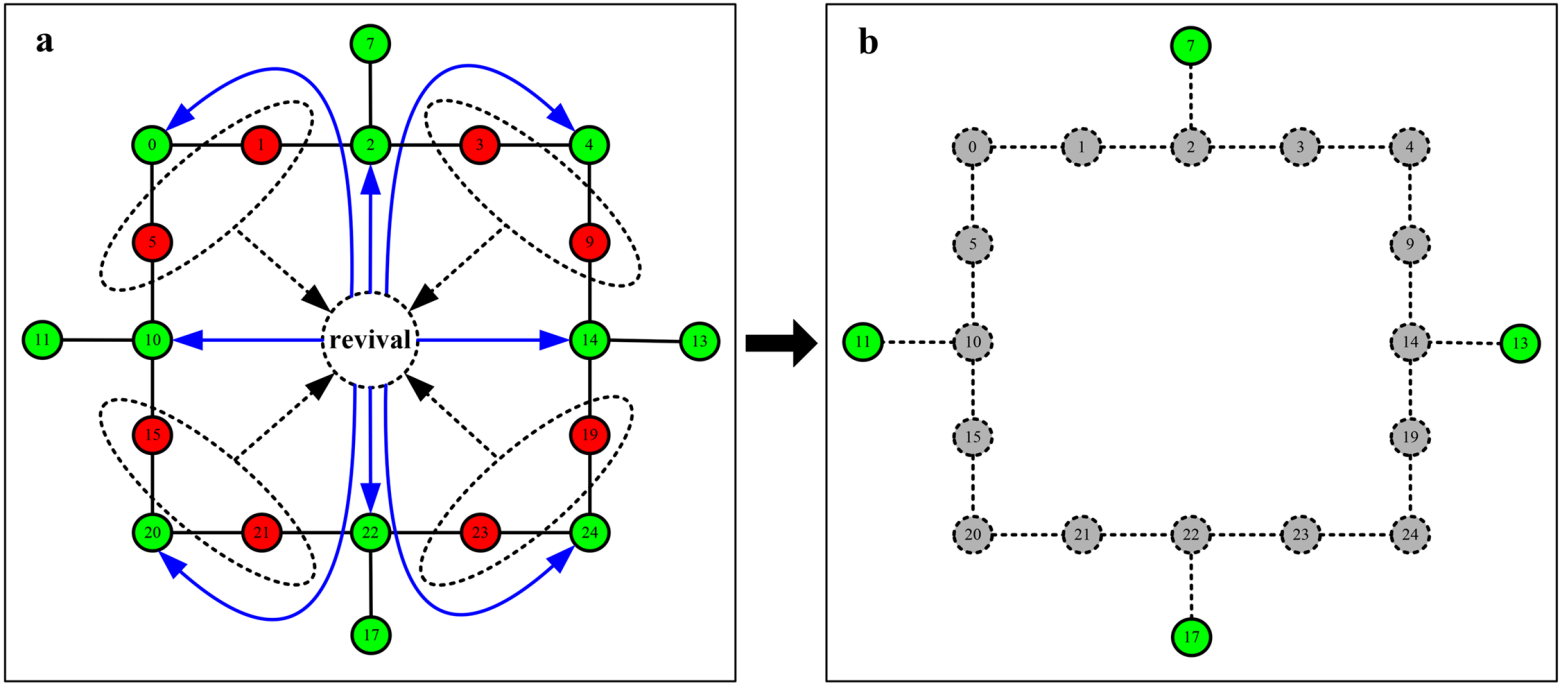
The scheme illustrates the reasonable explanation of the ability paradox in the cascading propagation by a simple illustration.

As the value of *β* increases from 0 to (15.34 − 11.2)/11.2, we observe a step by step process of the cascading propagation. We also find the counterintuitive phenomenon of the ability paradox at the critical point of *β* = (4.21 − 3.33)/3.33. What structure can lead to this phenomenon? To answer this question, in Fig 7, we carefully analyze the reason of the emergence of the oscillation of cascading dynamics. (a) The red nodes in the dotted line ellipse revive at this time (represented by the black dotted arrow). However, the revivals of these nodes immediately cause the failures of nine nodes connected with them (represented by the blue solid arrow). (b) Thus, the revivals of nodes inversely lead to a more catastrophic cascade which destroys the entire network. Therefore, we can summarize that the revivals of nodes in a network with the ring structure after removing nodes is the reason of the ability paradox in the cascading propagation.

## Conclusion

Considering the node weight and the preferential principle of the destination selection of the load transported, we introduce a new method to initialized loads distribution on nodes and propose a cascading load model. We focus on the cascading dynamics induced by removing the node with the highest initial load. In five synthetic networks, as the capacities of all nodes increase, we observe the oscillation of cascading dynamics. This finding is surprising, i.e., investing more resources to enhance nodes’ capacity inversely makes the whole network more vulnerable. To explain this ability paradox, we analyze step by step how cascading failures occur and how it propagates in a lattice network with 25 nodes and 40 edges. We find that this interesting phenomenon should be caused by the revivals of some nodes. By a simple illustration, we further summarize the underlying network structure that induces the observed turbulence, i.e., the revivals of nodes in a network with ring structures after removing nodes is the chief culprit of the ability paradox in the abnormal cascading propagation. Our findings should be useful in furthering studies in the control of cascade failures in real-world networks.

This work is only the first step towards understanding the behavior of cascading failures under the revised betweenness method. There are numerous aspects that require further investigation: which factors in the cascading modeling can affect the distribution of the initial load? Are there any effective methods to improve the robustness of a network? How should we solve the problem of the ability paradox in the cascading propagation? From the perspective of the betweenness method, how to construct the cascading model in the interdependent networks? We are planning to address these issues in our future work.
